# Safety of MST in clinical application: a systematic review of randomized controlled studies

**DOI:** 10.3389/fneur.2025.1539008

**Published:** 2025-08-21

**Authors:** Jinling Cheng, Zhanxiang Lin, Zicai Liu, Dongmiao Han

**Affiliations:** ^1^Department of Rehabilitation Therapy Teaching and Research, Gannan Healthcare Vocational College, Ganzhou, Jiangxi, China; ^2^Department of Rehabilitation Medicine, Shaoguan First People's Hospital, Shaoguan, Guangdong, China

**Keywords:** magnetic seizure therapy, MST, systematic review, safety, side effects

## Abstract

**Background:**

Magnetic seizure therapy (MST) is an innovative neurostimulation technique. While MST shares similarities with other neuromodulation techniques, such as electroconvulsive therapy (ECT) and transcranial magnetic stimulation (TMS), most research has predominantly focused on its efficacy. However, there is a notable scarcity of studies addressing MST’s safety. Therefore, the primary aim of this review is to synthesize the available safety data, contributing to a more balanced understanding of this promising treatment modality.

**Methods:**

Eight databases (PubMed, Embase, Cochrane Library, Web of Science, CNKI, CBM, VIP, and Wanfang Data) were searched up to August 2024. The evaluation process was strictly adhered to by the Cochrane Risk of Bias assessment tool and RevMan 5.4.

**Results:**

Of the 507 potential studies identified, after excluding duplicate studies, we reviewed the titles and abstracts of 217 studies, 149 of which were excluded. The full text of 68 studies was obtained and assessed against the inclusion and exclusion criteria, with 19 studies meeting the inclusion criteria. The therapeutic device and treatment parameters for MST are still being investigated, and the mechanism of MST is unclear, but there is almost consistent agreement on the efficacy and safety of MST.

**Conclusion:**

This study is the first systematic review of the safety of MST, and the findings suggest that MST can be used as an alternative treatment for certain psychiatric disorders with few side effects. Therefore, larger samples and more randomized controlled double-blind trials are needed in the future better to examine the clinical efficacy and safety of MST.

## 1 Introduction

**Figure 1:**
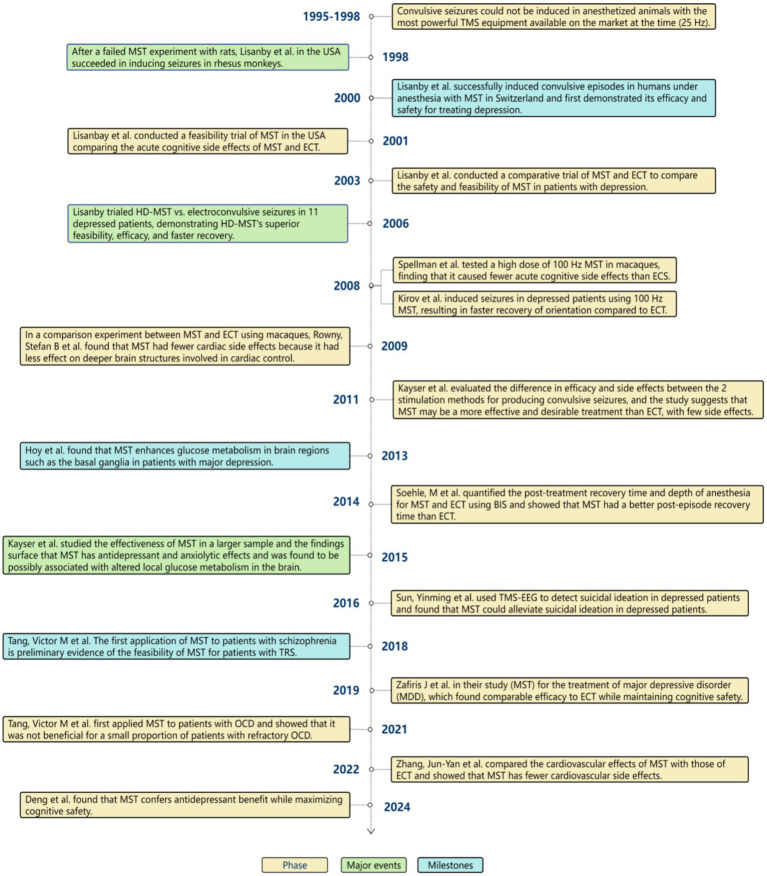
The development process of MST.

Magnetic seizure therapy (MST) is a non-invasive convulsive neurostimulation therapy (1, 2) that differs from ECT in that MST induces convulsions by sending magnetic stimulation (3) through high-frequency repetitive transcranial magnetic stimulation(rTMS) (4, 5), retaining the characteristics of limited, focused stimulation with high-frequency magnetic stimulation (6). In contrast, ECT uses direct electrical stimulation to induce convulsions. Dhuna et al. (7) were the first to show that magnetic stimulation might produce twitching when they conducted TMS tests in 1991. Lisanby et al. then conducted the first successful MST trials in non-human primates in 1998, followed by human MST experiments in 2000 (8). Trials on HD-MST started immediately after it was approved by the Food and Drug Administration (FDA) in the United States and numerous other industrialized countries. MST has presented a fresh approach to the treatment of psychiatric disorders, and more and more clinical studies have been done as a result of its high tolerability (9), minimal adverse effects, and general patient acceptability (10). We have now compiled a summary of the significant advancements in MST, as illustrated in Figure 1.

MST, a novel neuromodulation therapy (11), is as effective as traditional ECT but has fewer adverse effects (12, 13). Table 1 also shows us where MST has an advantage over ECT. Because MST is based on the principle that the magnetic field can pass unobstructed through the scalp and skull, as well as the fact that the magnetic field decays rapidly, the induced electric field has little effect on the scoficial layers of the cortex (14, 15), so MST is more targeted and focal to the site of stimulation than conventional ECT, avoiding direct stimulation of the medial temporal lobe structures (i.e., hippocampus) (16), resulting in a more focused and safer (17), MST offers a broader therapeutic range than ECT (making it more appropriate for elderly patients) and does not have the cognitive and memory adverse effects that ECT does (18). MST outperforms conventional instrument management approaches by combining automation, interoperability, and data intelligence to create a resilient, future-ready operational framework. Its emphasis on integration, scalability, and cost-effectiveness makes it a transformative solution for modern industrial and laboratory settings. Recent research shows that MST improves treatment results in psychiatric diseases such as major depressive disorder, bipolar depression, and schizophrenia (12) and that MST also decreases anxiety and increases the quality of life while improving neurological and cognitive areas (19). There are of course contraindications to MST, as described in Table 2, and it should not be used in patients with implanted electronic devices, pregnancy, dementia, etc.

**Table 1:** Technical advantages of MST over ECT.

Point	MST	ECT
Electric current	The induction current runs parallel to the cerebral cortex, making it difficult to reach the deeper layers of the brain.	Stimulation current perpendicular to the cerebral cortex
Brain effects	Little or no impact	Alters the structure of the hippocampus in the deep part of the brain
Adverse effects	Myotonia, headache, dizziness(Less)	Causes adverse effects such as memory and cognitive impairment, and bradycardia(More)
Cognitive side effects	Less	More
Directional function recovery	Quick	slow
Attention deficit	Less	More
anterograde and retrograde amnesia	Less	More
Induction and transmission of localized seizure	Strong	weak
Operability	Good	Normal
Tolerance	Good	Normal
Patient Acceptance	Good	Normal

Normal: Refers to a situation where patient acceptance and tolerance is not very good due to considerations such as knowledge of the device and side effects.

**Table 2:** Contraindications to MST.

No	Contraindications
1	The presence of a ferromagnetic metal foreign body or electronic device built into the skull
2	Patient with pacemakers, heart stents
3	Patient with cochlear implants
4	Those with significantly increased intracranial pressure
5	Patient with allergies to anesthetics
6	Patient with severe physical illnesses that cannot tolerate the therapy (Severe infections, brain-occupying diseases, severe somatic diseases, pheochromocytoma and severe endocrine diseases, glaucoma, retinal detachments)
7	Dementia, delirium, amnesia
8	Severe head trauma
9	Stroke, seizure, multiple sclerosis
10	Drug dependence
11	Heart attack, liver failure, tumors, immunodeficiency
12	Pregnancy
13	Unable to participate in clinical and psychological testing

MST is an effective treatment for depression in the majority of studies to date, with over 50% of patients responding to MST for depression in one study (19), treatment efficacy rates of 40–70%, and depression remission rates of 15-46EEG% (8), and improved tolerability of MST may make patients more supportive of acute and long-term maintenance programs (20). As a result, if more research on the effects of MST on cognitive function in the treatment of depression is conducted in the future, MST has the potential to become the first choice for antidepressant physiotherapy (18, 21), but there may still be adverse effects due to general anesthesia and convulsions during MST treatment (22). Although a recent study by Jiang, Jiangling et al. noted that the safety of MST has been well documented in animal studies and human studies (23), there is no systematic review of the safety of MST in the treatment of various diseases and applications, and there is a need for a comprehensive evaluation based on the evidence related to the safety of MST.

The purpose of this paper is to provide a systematic review of the safety aspects of MST, which will provide a solid foundation for future clinical applications of MST.

## 2 Materials and methods

### 2.1 Search strategy

We used Chinese databases such as CNKI, CBM, VIP, and Wanfang, as well as English databases such as PubMed, Cochrane Library, Embase, and Web of Science, to conduct a systematic search. These databases are combed through to August 2024. The keywords “MST,” “magnetic seizure technique,” and “safety” were used to search for studies. References to these studies were also searched to find all potentially optimal studies.

### 2.2 Search terms

“Magnetic seizure therapy” and “safety or security.” Using the PubMed database as an example, the search strategy was as follows (Table 3).

**Table 3:** The specific search strategy of the Pubmed database.

No.	Search items	Results
#1	“Magnetic seizure therapy” [Title/Abstract]	175
#2	“safety” [Title/Abstract]	770,741
#3	“Security” [Title/Abstract]	78,941
#4	#2 or #3	770,741
#5	#1 and #4	33

### 2.3 Eligibility criteria

Our systematic review includes any trials on the safety of MST. The following were the specific inclusion criteria: (1) patients with any disease; (2) MST as an intervention; (3) no control, experimental (any treatment), and control (placebo or no treatment) conditions; (4) the magnitude of side effects after MST treatment, recovery time after treatment, and physical condition after treatment were the main indicators. The following were the exclusion criteria: (1) missing articles or data; (2) significant bias risk; (3) duplicate literature; (4) reviews, conference abstracts, and case studies; (5) enumerating significant bias risks.

### 2.4 Study selection and data extraction

First, the literature that was found was imported into EndNote’s literature management system. Second, titles and abstracts were strictly screened by the inclusion and exclusion criteria. After an initial screening based on the title and abstract, all articles possibly relevant to the study were downloaded and viewed. After reading the full text, the corresponding articles were included. During the screening process, disagreements were resolved through discussion or by another person.

Two researchers extracted data from the included studies independently. First author, year of publication, sample size, gender, mean age, duration of illness, site of action, intervention method, duration of intervention, and outcome measures were all extracted. The Hamilton Depression Scale was the primary indicator (HAMD or HDRS).

### 2.5 Quality assessment

Two researchers independently assessed the quality of the included studies using the Cochrane Risk of Bias tool (Revman 5.4). The risk of bias was evaluated in seven areas: whether the random allocation was used, whether the intervention allocation was concealed, whether participants and investigators were aware of the grouping, whether outcome data were complete (someone dropped out halfway through), whether the assessor’s confidentiality was maintained, and whether there was selective reporting and other sources of bias (e.g., treatment criteria, adverse events, etc.). To address controversial matters, consult with a third expert investigator if necessary.

## 3 Results

### 3.1 Study selection

**Figure 2:**
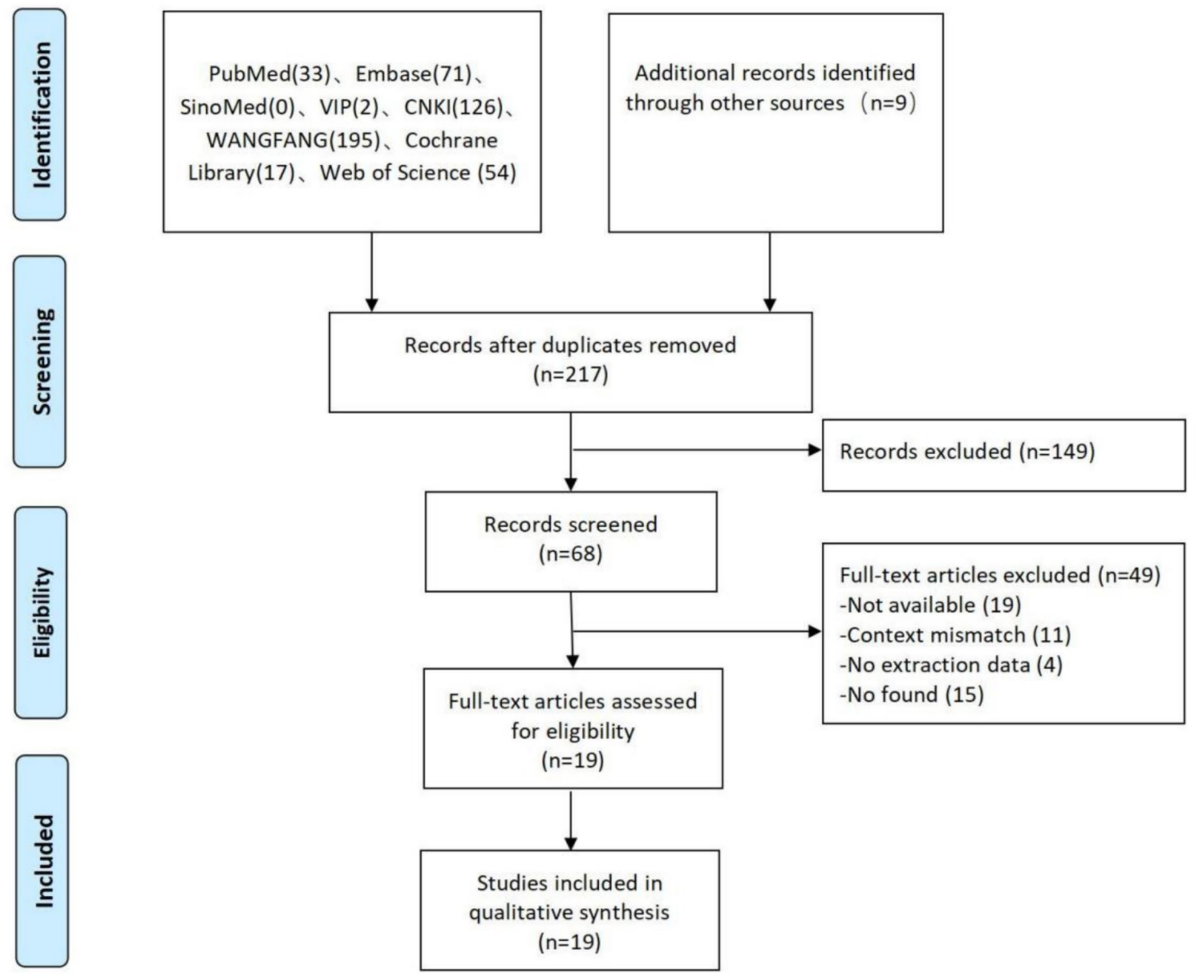
Flowchart of the study search and selection process.

The first search generated a total of 507 articles. After filtering by reading titles, abstracts, and duplicates, 149 studies were excluded and 68 articles were selected for a reading of the full text. Of these, 19 articles met the inclusion criteria, as illustrated in Figure 2.

### 3.2 Study characteristics

#### 3.2.1 MST stimulation site and parameters

The basic characteristics of the 19 selected studies are shown in Tables 4, 5. In these studies, the devices were mainly provided by the companies Magstim and Magventure. For the placement of the MST, most treatments were performed using vertex stimulation (15 studies), Yinming Sun et al. (24) chose to stimulate the frontal area, and three studies did not specify the site of stimulation (22, 25, 26). Stimulation parameters also differed, with most of these treatments choosing low-pulse treatments, but few studies specifically reported stimulation intensity, and stimulation frequency, except the earliest in 2003, which set a frequency of 40–60 Hz (10), most studies designed MST at 100 Hz, two set it at 50 Hz (22, 23), two set it at 25–100 Hz (27, 28), and one did not report a frequency (24). The duration of action ranged from 0.3s to 20s, with an average of 2–3 treatments per week and an average length of time ranging from 2 weeks to 6 weeks (29).

**Table 4:** The detailed features of the studies.

Study	Country	Diagnostic criteria	Sex ratio (F/M)		Age mean (year)		Course of disease		Primary outcome
			Test group (MST)	Control group (ECT)	Test group (MST)	Control group (ECT)	Test group (MST)	Control group (ECT)	
Fatma A. El-Deeb (48)	America	DSM-IV	17\13	BT:8\7 RUL:5\10	39.07 ± 12.85	BT:38.80 ± 14.0 RUL:39.6 ± 12.32	7.73 ± 5.66 m	BT:6.00 ± 5.42 m RUL:5.73 ± 4.03 m	HAMD-21, BDI
Kayser (49)	Germany	DSM-IV	6\4	7\3	48.80 ± 8.35	52.8 ± 11.43	6.01 ± 10.42y	3.5 ± 4.12y	MADRS, HDRS28
Fitzgerald (52)	Australia	HAMD > 18, DSM-IV	8\10	13\6	44.6 ± 14.8	47.2 ± 16.1	22.7 ± 14.3y	27.6 ± 14.4y	HAMD17
Polster (50)	Germany	HDRS-28 ≥ 20	3\7	6\4	43.7 ± 11	54.7 ± 16.1	4.1 ± 4y	3.1 ± 3y	HDRS28, BDI
Kayser (19)	Germany	HAMD28 ≥ 20	14\12	\	47.2 ± 10.0	\	14.9y	\	HAMD28, HAMD17
Lisanby (10)	America	DSM-IV	7\3	7\3	46.77 ± 10.0	46.77 ± 10.0	\	\	HDRS24
Sarah Kayser (53)	Germany	HDRS28	4\6	\	42.1 ± 10.0	\	\	\	HDRS28, MADRS
Fitzgerald (29)	Australia	MADRS>20	10\3	\	46.77 ± 14.82	\	17.31 ± 9.61y	\	MADRS
Victor M. Tang (27)	Canada	DSM-IV HDRS-24 ≥ 21	17\9	\	47.30 ± 14.23	\	113.35 ± 111.74 W	\	HDRS24
Hoy (25)	Australia	MADRS>20	8\2	\	44.1 ± 14.36	\	\	\	MADRS
Jiangling Jiang (23)	France	DSM-5 PANSS≥60	24\19	22\14	31.3 ± 9.3	33.8 ± 10.8	8.0 ± 7.0y	7.8 ± 6.8y	PANSS
Junyan Zhang (16)	China	HAMD-17>17	16\2	22\5	29.00 ± 8.32	32.78 ± 8.84	3.77 ± 3.77y	4.47 ± 5.42y	HAMD-17, HAMA
Kayser (51)	Germany	DSM-IV	2\5	2\5	43.43 ± 5.59	43.43 ± 5.59	6.29 ± 6.04y	6.29 ± 6.04y	HDRS28, MADRS, BDI
Kayser (26)	Germany	DSM-IV	3\7	4\6	45 ± 14	55 ± 12	\	\	HDRS28
Kirov (35)	UK	DSM-IV	8\3	8\3	42.64	42.64	\	\	\
Sravya Atluri (28)	Canada	DSM-IV	12\12	14\8	42.0 ± 13.4	46.8 ± 15.8	20.3 ± 13.7	19 ± 12.0	HDRS, MADRS, BDI
White (22)	America	\	6\4	6\4	48 ± 4	49 ± 6	\	\	HAMD-17
Yinming Sun (24)	Canada	DSM-IV	12\11	\	45.0 ± 12.2	\	20.7 ± 15.0	\	HDRS24, SSI
Deng (30)	America	DSM-IV-TR	19/19	22/13	48.2 (12.8)	47.7 (15.6)	114.5 (129.4) w	135.2 (208.3) w	HDRS-24

MST, Magnetic seizure therapy. ECT, Electroconvulsive therapy. DSM-IV, diagnostic and statistical manual of mental disorders. HDRS, Hamilton depression rating scale. HDRS-24, 24-item Hamilton depression rating scale. HDRS-28, 28-item Hamilton depression rating scale. HAMD, Hamilton Depression Rating Scale. HAMD17, 17-item Hamilton Depression Rating Scale. HAMD21, 21-item Hamilton Depression Rating Scale. HAMD-28, Hamilton Depression Rating Scale-28. MADRS, Montgomery-Asberg depression rating scale. BT, bitemporal. RUL, right unilateral. PANSS, the Positive and Negative Syndrome Scale. BDI, Beck Depression Inventory. SSI, the Scale for Suicidal Ideation.

**Table 5:** Main parameters of MST.

Study	Interventions		Time	Secondary outcome	Drop-outs due to side effects/other reasons	Device	Corporate brand
	Test group (MST)	Control group (ECT)					
Fatma A. El-Deeb (48)	100 Hz 10s MST	0.5 msec BT-ECT RUL-ECT	2 t/w, 2.5w	TRO	0	Magstim Theta	Magstim
Kayser (49)	100 Hz 6 s 0.37 msec MST	4–8 s 0.5 msec RUL-ECT	2 t/w, 6w	AMI	0	MagPro MST	MagVentureA/S, Denmark
Fitzgerald (52)	100 Hz 2–10s MST	1.0 msec RUL-ECT	3 t/w, 4–5w	AMI	0/3	Magventure	Magventure A/S (Denmark)
Polster (50)	100 Hz 5–8 s MST	5–8 s 0.5 msec RUL-ECT	2 t/w, 5-6w	Likert scale	0	MagPro MST	Magventure A/S, Farum, Denmark
Kayser (19)	100 Hz 10s 0.2 msec MST	\	2 t/w, 2-11w	MMSE	0	MagPro MST	Magventure A/S, Denmark
Lisanby (10)	40-60 Hz 0.5–8.0 s MST	0.5 msec BL-ECT RUL-ECT	3 t/w	NPB	0	Magstim Theta	Magstim
Sarah Kayser (53)	100 Hz 6.6 s 0.28 msec MST	\	\	AMI	0/1	MagPro MST	Magventure A/S, Denmark
Fitzgerald (29)	100 Hz 10s MST	\	3 t/w, 2-6w	AMI	0/1	\	Magventure A/S and Brainsway Ltd
Victor M. Tang (27)	25/50/60/100 Hz MST	\	2-3 t/w	AMI	0/6	MagPro MST	Magventure
Hoy (25)	100 Hz 10s MST	\	3 t/w, 2-6w	AMI	0	\	Magventure A/S
Jiangling Jiang (23)	50 Hz 4–20s MST	0.5 msec ECT	2-3 t/w,4w	RBANS	4\6	MagPro MST	Magventure A/S, Denmark
Junyan Zhang (16)	100 Hz 10s MST	BL ECT	6 times	RBANS	0	Magstim Inc.	
Kayser (51)	100 Hz MST	0.5 msec BL-ECT RUL-ECT	2 t /w, 6w	Recovery and reorientation times	0	MagPro MST	Magventure A/S, Denmark
Kayser (26)	100 Hz 6.5 s MST	0.5 msec BL-ECT RUL-ECT	2 t/w, 6w	\	1\4	MagPro MST	Magventure A/S, Farum, Denmark
Kirov (35)	100 Hz 10s MST	BT-ECT RUL-ECT	\	Recovery of orientation	0	Magstim Theta	Magstim
Sravya Atluri (28)	25/50/60/100 Hz MST	BT-ECT RUL-ECT	\	MoCA	0	MagPro MST	Magventure
White (22)	50 Hz 8 s MST	0.5 msec ECT	3-4 t/w, 3-4w	Recovery time	0	Magstim	Magstim Co. LTD, Wales, United Kingdom
Yinming Sun (24)	\	\	24 times	\	0	MagPro MST	Magventure
Deng (30)	100 Hz, 10s, MST	RUL ECT	3 t/w,>8 t,(HDRS-24 ≤ 8)	Depressive Symptomatology	14/6	Magstim Theta	spectrum 5000Q or Thymatron System IV

MST, magnetic seizure therapy. ECT, electroconvulsive therapy. MMSE, mini-mental state examination. NPB, neuropsychological battery. AMI, autobiographical memory interview. BT, bitemporal. RUL, right unilateral. MoCA, Montreal Cognitive Assessment. RBANS, Repeatable Battery for the Assessment of Neuropsychological Status. TRO, time to reorientation. T, times. W, weeks.

#### 3.2.2 Outcomes

**Figure 3:**
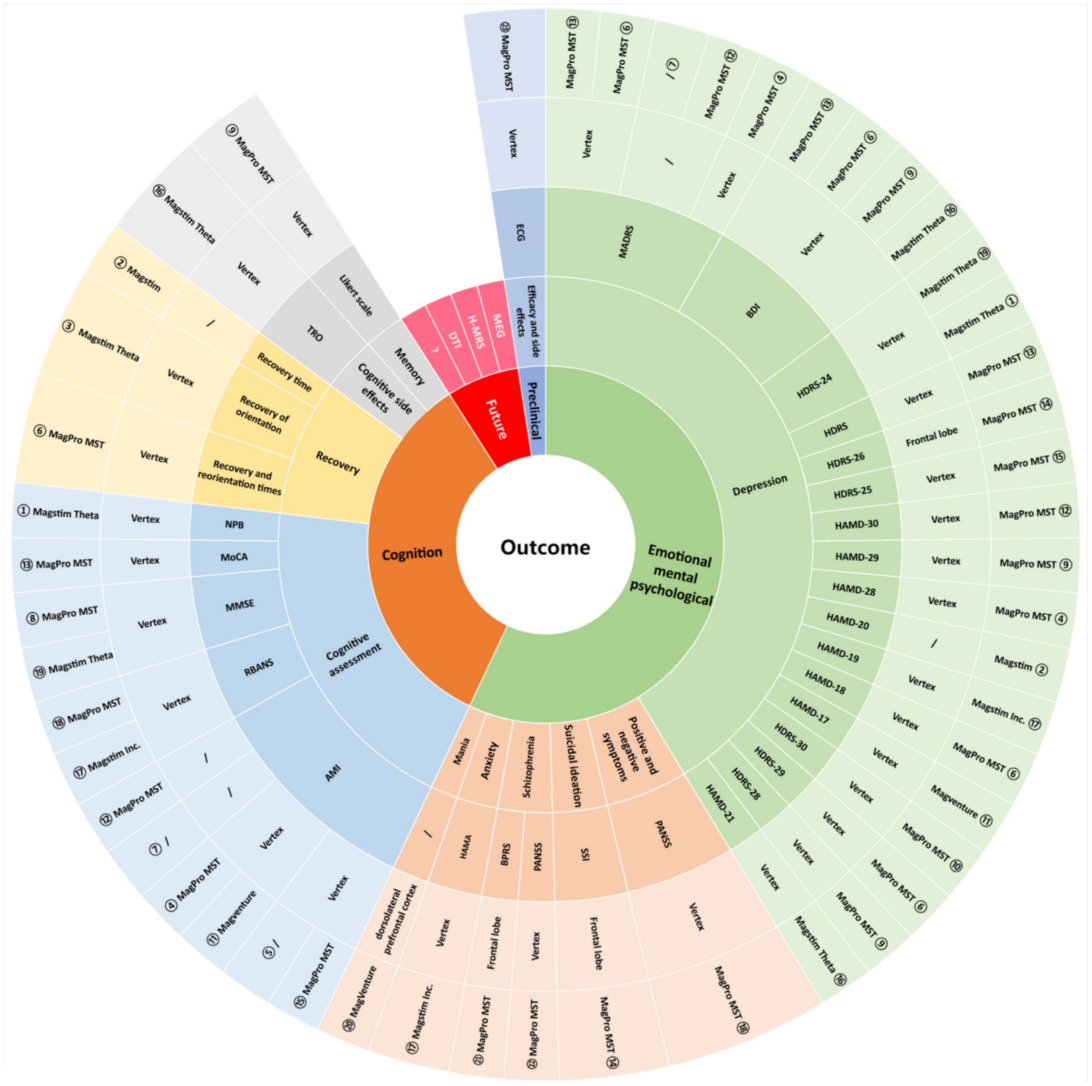
Outcome characteristics of the included studies. ① Lisanby (10); ② White (22); ③ Kirov (35); ④ Kayser (49); ⑤ Fitzgerald (29); ⑥ Kayser (51); ⑦ Hoy (25); ⑧ Kayser (19); ⑨ Polster (50); ⑩ Kayser (26); ⑪ Fitzgerald (52); ⑫ Kayser (53); ⑬ Atluri (28); ⑭ Sun (24); ⑮ Tang (27); ⑯ Deeb (48); ⑰ Zhang (16); ⑱ Jiang (23); ⑲ Deng (30); ⑳ Noda (45); ㉑ Tang (37); ㉒ Li (21); ㉓ Peterchey (55).

The primary outcome indicator in the included studies was the clinical outcome, and the secondary outcome indicator was a change in cognitive function. Scales such as the HAMD, HDRS, and PANSS were used to assess the efficacy of the MST, and all studies showed an improvement in patients’ symptoms following the application of the MST for treatment, which Hoy et al. (25) Suggest may be related to the increased metabolism of brain-related structures. Scales such as TRO, AMI, and MMSE were used to assess cognitive function in patients after treatment, with two studies noting no cognitive side effects from MST and eight studies showing faster recovery of cognitive function after treatment than ECT. White et al. (22) found this may be related to the fact that MST requires fewer muscle relaxants and reduces the variability of BIS values after epilepsy induction. In addition, we made an in-depth summary of the application of MST in preclinical research and future development. A detailed summary of MST-related research outcomes (including symptoms, assessment indicators, targets, equipment, etc.) is shown in Figure 3.

#### 3.2.3 Side effects

The most common side effects of MST are myotonia, pain, and impaired cognitive function, but no specific side effects such as myotonia or pain have been reported. Five patients withdrew from treatment midway through the course of treatment due to side effects (23, 26), all of whom returned to normal after stopping treatment. There were four adverse events in 482 subjects, two of which were thought to be possibly related to MST (27), one of which developed manic symptoms after treatment but recovered after increasing the medication, and the other dislocated his shoulder after a fall and resumed the trial after stabilization, 2 patients reported nausea and vomiting after MST treatment (30). But otherwise, there were no adverse events regarding MST.

### 3.3 Risk of bias

**Figure 4:**
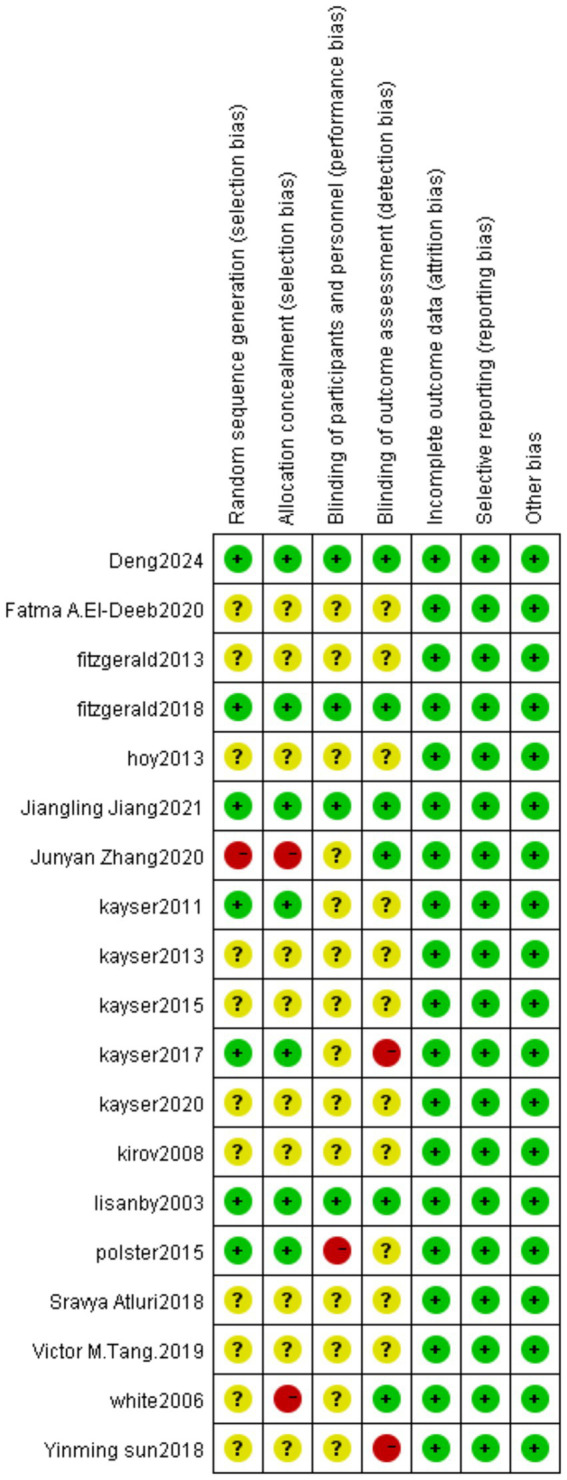
Risk of bias summary: review authors’ judgments about each risk of bias item for each included study.

**Figure 5:**
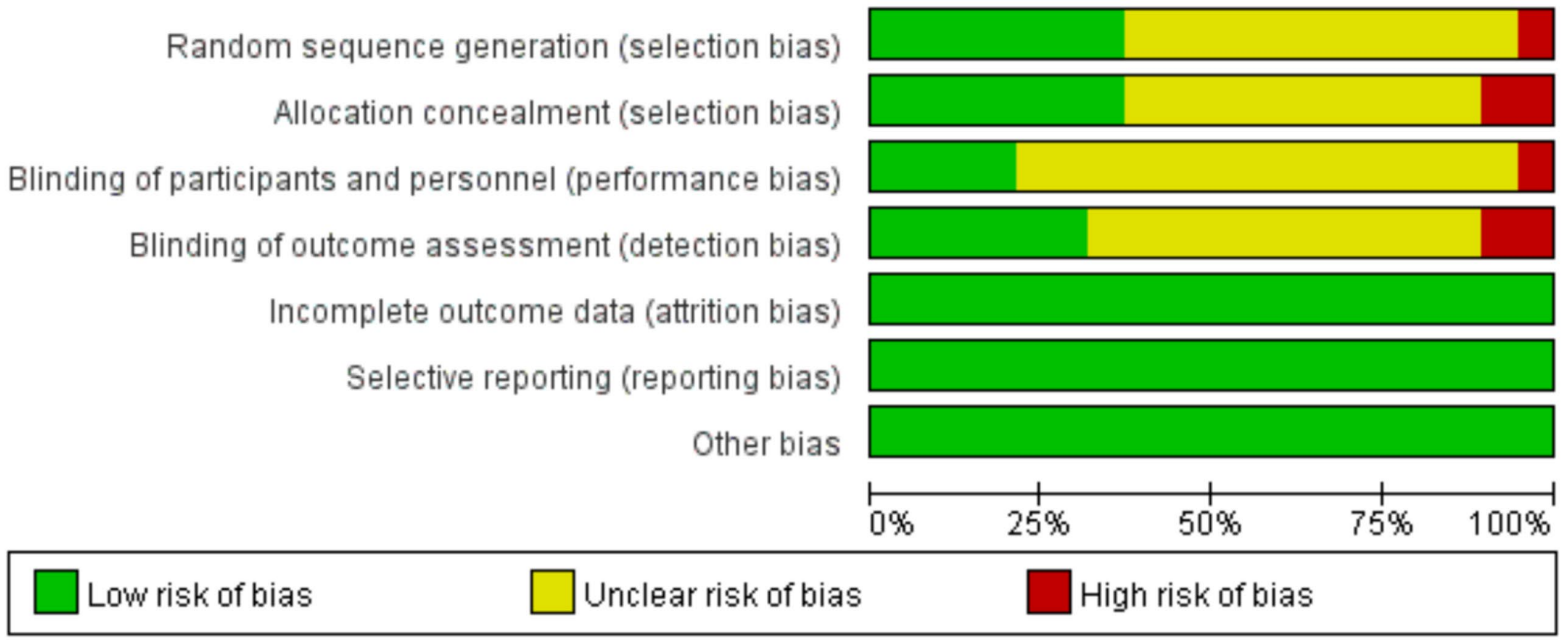
Risk of bias graph: review authors’ judgments about each risk of bias item presented as percentages across all included studies.

We assessed the risk of bias for included studies according to the Risk of Bias Assessment table of the Cochrane Manual of Systematic Reviews, version 5.4. The results of bias risk are shown in Figures 4, 5. Seven of the 19 studies described randomization as a method. Only four studies described the blinding method for the participants, six studies described the blinding method for the evaluators, and the other studies did not mention the blinding method. Probably because the MST is a device that produces effects by stimulating the brain, most studies are open-label experiments, which increases the risk of bias. Overall, all of the studies require some attention.

## 4 Discussion

MST is a technique that has been developed steadily since 1991. MST got increased attention, owing to the common perception in the medical profession at the time that tics might treat various mental disorders, and the necessity to tap into new approaches due to the limits of some of them. Lisanby applied MST on people for the first time in 2000, and this experiment also showed for the first time that MST was safe and practicable in treating depression (10). Since then, researchers have been examining the safety and efficacy of MST in treating mental diseases like depression. ECT has long been the main antidepressant treatment choice (29, 31, 32); however, many negative ECT outcomes (33, 34) have damaged its credibility, necessitating the urgent need for a new technology to replace it. Following several clinical trials, researchers discovered that MST was equally effective at treating depression as ECT, with quicker post-treatment recovery times for memory and orientation (35, 36), and no cognitive side effects. As a result, some researchers have since suggested MST as an alternative to ECT (37).

MST’s mechanisms, like those of ECT, have not been clarified as a combination of TMS and ECT. Because MST and ECT are both convulsive procedures, the processes may overlap, and existing ideas on the prevalence of ECT include neuroendocrinology, neuronal cell plasticity, and brain plasticity in seizure (38, 39); thus, MST mechanisms may apply to these as well. With the advancement of science and technology, additional research has been undertaken on MST. MST is currently recognized to ease depressive symptoms and reduce suicidal thoughts in depressed patients (40), and it has also been utilized in therapies for diverse illnesses such as schizophrenia (41), obsessive-compulsive disorder (12), and bipolar disorder (42). MST has been well demonstrated in these experiments to be a relatively effective and safe technique. This may be due to its role as a unique and experimental therapeutic procedure that combines the efficacy of ECT with the safety of TMS (43). However, because MST is based on ECT and other techniques (44), there are some potential safety hazards of the fundamental method in its use, such as causing memory loss, cognitive impairment, and mania (45); thus, the safety of MST must be evaluated. The goal of this study was to thoroughly examine published trials on MST safety to summarize the evidence supporting MST safety in its application and give a foundation for treatment possibilities for various disorders.

This study found that the efficacy and safety of MST have been demonstrated in the treatment of several psychiatric disorders. The 18 studies we included in total contained 482 subjects, of which two serious adverse events were considered to be possibly related to MST (both from the same study) (19). In addition, some studies reported side effects of MST, but these were mostly due to anesthesia-induced myotonia, so few participants dropped out of the study because of side effects. Most of the experiments used EEG (46) to monitor patients, with four studies highlighting the data observed by EEG: for example, the original Kirov et al. study found that the MST only matched patients when the coil was placed above the vertex and the seizure threshold was below the prefrontal or central anterior mass (35) [It was not until 2016 that sun et al. et al. found that stimulation of the dorsal frontal cortex may produce a better response (47)]; when kayser et al. used BIS to monitor individuals to determine the timing of seizure induction, they found that both treatments, MST and ECT, resulted in more pronounced post-seizure suppression (26); Yiniming sun et al. observed that MST can produce neuroplasticity in the frontal cortex (24); Altluri et al. demonstrated for the first time that the resting state can be regulated by seizure treatment in patients with TRD (28). Only Kayser et al. showed that the other 18 responders were followed up for 6 months after the trial and were analyzed in detail, finding that patients mostly relapsed 2–3 months after treatment (19). This demonstrates the relatively long-lasting relief effect of MST. Combining these 18 studies, in terms of safety, the results of three studies clearly indicate that MST is a safe technology and also confirm its feasibility (19, 27, 48); in terms of therapeutic mechanisms, hoy et al. found that MST not only improved depressive symptoms, but also found that this may be related to the fact that MST increased the metabolic rate of local brain tissue (25); in terms of the underlying data for MST, fizt2012 et al. suggested that appropriate parameters could be selected after 10 s of continuous stimulation; finally, in a controlled trial of MST and ECT, the researchers found that MST was comparable to ECT in efficacy and had fewer cognitive side effects (16, 23, 48–51) [Only in the Fitzgerald et al. trial could MST not be shown to be as effective as ECT (52)]. In addition, Lisanby et al. showed that the MST group outperformed the ECT group in orientation recovery, attention, anterograde and retrograde amnesia (10), and even in one study kayser et al. stated that MST does not lead to anterograde and retrograde amnesia (53).

The study’s strength is its thorough description of the safety aspects of MST treatments in various illnesses and experimental subjects, which allows us to evaluate the safety of MST objectively and systematically, owing to the extensive search. The study’s results suggest that MST is well-tolerated and safe in humans. Memory loss and myotonia were the most common side effects, but they all resolved quite rapidly after stopping therapy, and there were few major adverse events. It improves when treatment is stopped or medication is increased. The study also has shortcomings. First, the included studies predominantly featured small sample sizes (median *n* = 26 per trial), which limits statistical power for detecting rare adverse events. Second, significant heterogeneity in MST parameters complicates cross-study comparisons. Third, only 7/19 studies described randomization methods, and fewer than 30% implemented participant/assessor blinding—a limitation inherent to device-based trials where sham controls are challenging. Finally, 16/19 studies lacked follow-up beyond 6 months, preventing long-term safety assessment. Prospective RCTs with standardized protocols, active/sham controls, and multi-year follow-ups are urgently needed to establish MST’s safety profile. MST has been investigated for about 30 years (since 1995) and is difficult to expand in the clinic because of its costly equipment needs (54), therefore, future trials might be started with new frequencies and stimulation modalities and employed in additional disorders to explore MST’s efficacy. Consideration should also be given to the effectiveness of MST in multicultural and multi-ethnic environments. Success requires balancing technical standardization with cultural sensitivity, ethical transparency, and inclusive design. Failure to take these differences into account may exacerbate inequalities or lead to exclusion from the system.

## 5 Conclusion

In conclusion, the results of this study suggest that MST is a safe neuromodulation technique, with some studies suggesting that the stimulation patterns and stimulation ranges of MST and ECT are different, which may account for the different magnitudes of side effects they produce. This is why more clinical studies and basic experiments using MST in more diseases and species are needed in the future to investigate its mechanism of action and to fully show its efficacy and safety. Such trials will contribute to the development of the neurotherapeutic field and help to confirm and establish MST as a safe and proven therapeutic technique for the treatment of severe mood disorders. While MST demonstrates short-term safety and tolerability in current evidence, its clinical adoption requires larger, rigorously designed trials addressing methodological gaps identified herein.

## Data Availability

The original contributions presented in the study are included in the article/supplementary material, further inquiries can be directed to the corresponding authors.
